# A novel duodenal-release formulation of caraway oil and L-menthol is a safe, effective and well tolerated therapy for functional dyspepsia

**DOI:** 10.1186/s12876-022-02181-5

**Published:** 2022-03-07

**Authors:** Brian E. Lacy, William D. Chey, Michael S. Epstein, Syed M. Shah, Patrick Corsino, Linda R. Zeitzoff, Brooks D. Cash

**Affiliations:** 1 Gastroenterology and Hepatology, Mayo Clinic, 4500 San Pablo Road South, Jacksonville, FL 32224 USA; 2 Division of Gastroenterology, Department of Internal Medicine, Michigan Medicine, University of Michigan, Ann Arbor, MI 48109 USA; 3 Digestive Disorders Associates, 621 Ridgely Ave, #201, Annapolis, MD 21401 USA; 4 Nestlé Health Science, 1007 US Highway 202/206, Building JR2, Bridgewater, NJ 08807 USA; 5 IM Health Science, 1100 Holland Drive, Boca Raton, FL 33487 USA; 6 Medical Affairs, Nestlé Health Science, 1007 US Highway 202/206, Building JR2, Bridgewater, NJ 08807 USA; 7 Division Gastroenterology, Hepatology and Nutrition, McGovern Medical School, University of Texas Health Science Center at Houston, Houston, TX 77031 USA

**Keywords:** Abdominal pain, Caraway oil, Dyspepsia, Functional dyspepsia, L-menthol

## Abstract

**Background:**

A randomized, placebo-controlled clinical trial (FDREST) of a novel formulation of caraway oil and L-menthol (COLM-SST) demonstrated symptom relief in patients with functional dyspepsia (FD). Two follow-up studies were conducted to evaluate patient satisfaction, self-regulated dosing, and long-term safety data: FDACT, Functional Dyspepsia Adherence and Compliance Trial, and FDSU36, Functional Dyspepsia Safety Update at 36 months.

**Methods:**

A patient reported outcomes (PRO) questionnaire was designed and distributed online to assess real-world satisfaction and dosing frequency of open-label COLM-SST in patients with FD. A separate study analyzing voluntary safety surveillance data evaluated the frequency and severity of reported adverse events (AEs).

**Results:**

A total of 600 FD patients were enrolled in the PRO study. Ninety five percent of respondents reported a major or moderate improvement in their FD symptoms and 91.7% indicated a major or moderate improvement in quality of life (QOL) using COLM-SST. Between 1 and 4 capsules were consumed daily by 91.2% of respondents, with 56.2% taking them before meals. Symptom relief was rapid, with 86.4% of respondents indicating relief within 2 h of taking COLM-SST. Few adverse events (AEs) were reported (0.0187%) by patients using COLM-SST. No serious AEs were identified.

**Conclusion:**

COLM-SST is safe, well tolerated, and provides rapid relief of FD symptoms. These findings, demonstrated in the FDREST trial, were further supported by a large prospective PRO study evaluating self-regulated dosing frequency, symptom improvement, and QOL. COLM-SST was well-tolerated based on review of AE data at 36 months.

## Background

Functional dyspepsia (FD) is a disorder of gut-brain interaction (DGBI) that affects the upper portion of the gastrointestinal tract. The disorder is more frequently diagnosed in women than men [1]. While the prevalence of FD varies among countries and regions, it is estimated that the disorder affects up to 16% of otherwise healthy individuals in the general population [2–5]. Symptoms of FD may include epigastric pain, epigastric fullness or pressure, early satiation, nausea, and bloating. Functional dyspepsia has been described as a “constellation of symptoms” that vary in intensity and frequency from patient to patient [6]. These symptoms can be triggered by food intake and generally increase in severity as the meal progresses with an eventual return to baseline [7, 8]. A longitudinal natural history study demonstrated that, for many patients, recurrent FD symptoms can persist for years [9]. Functional dyspepsia can be diagnosed after a careful history and physical examination along with limited diagnostic tests to exclude an organic disorder. The Rome criteria (Rome IV) separates FD into two broad symptom-based subgroups—epigastric pain syndrome (EPS) and postprandial distress syndrome (PDS) [10]. Epigastric pain syndrome is characterized by recurrent epigastric pain and burning, while PDS is characterized by postprandial fullness/early satiety and the sensation of bloating [11]. Although it was hoped that this classification system would clearly differentiate FD subgroups based on physiology, thus allowing individualized treatment choices, this has not proved true, as investigations have failed to identify unique pathophysiologies between the two FD subgroups [12, 13]. Symptoms of FD can be quite bothersome and have significant negative impact on quality of life and meal quantity and quality [14]. The negative economic impact of FD is striking. One study estimated that the extra cost for treatment as well as reduced work hours from FD symptoms totaled more than $18 billion per year in the United States. [15].

While no medication is approved for the treatment of FD, physicians prescribe a variety of treatments in off-label fashion to patients with FD to improve their symptoms. This approach has met with varying degrees of success. Commonly used classes of medications for FD include, but are not limited to, histamine type 2 receptor antagonists (H2RAs), proton pump inhibitors (PPIs), prokinetic agents, tricyclic antidepressants, selective serotonin reuptake inhibitors (SSRIs), and selective serotonin and norepinephrine reuptake inhibitors (SNRIs) [16]. While some of these therapies have shown beneficial effects, others have been linked to significant safety concerns. A recent review of prokinetic agents suggests some symptom benefit for both subgroups of FD. Apart from cisapride, medications commonly used for FD appear to be well-tolerated for short-term treatment [17]. Cisapride, a 5-HT_4_ receptor agonist, was removed from the market due to safety concerns related to QT prolongation. [18]. Metoclopramide, a prokinetic agent used for many years, is associated with the risk of tardive dyskinesia and other neurological symptoms [19]. Taking into account known risk factors among metoclopramide users such as advanced age, antipsychotic drug therapy, and patients with renal and hepatic insufficiency, the risk is estimated to be approximately 0.1% per 1000 patient years [20]. Proton pump inhibitors may improve symptoms in some patients although the number needed to treat (NNT) to achieve symptom relief is fairly high at 10–14 [21]. Patients and providers have continued to express the desire for safe and efficacious treatments for FD. [18].

As an alternative to using off-label prescription drugs, non-prescription therapies, including herbal-based treatments, have been used by patients and health care providers to address the multiple symptoms of FD. The combination of peppermint oil and caraway oil has been studied in a number of clinical trials and has consistently been shown to be effective at treating FD symptoms [22–24]. One study showed comparable efficacy between a peppermint oil/caraway oil combination and cisapride [25]. More recently, a clinical study was completed using a novel-release combination of caraway oil and L-menthol (COLM-SST), the key active ingredient of peppermint oil, with a microsphere-based site-specific targeting delivery system [26]. This unique delivery system was designed to allow the active ingredients to be released at what is considered the site most affected in many FD patients, the duodenum [27, 28]. In addition, release of L-menthol beyond the pylorus is hypothesized to decrease the incidence of L-menthol induced gastroesophageal reflux symptoms. Results from a 4-week, randomized, controlled trial were favorable, indicating that COLM-SST could improve symptoms of FD and was well tolerated. [26].

To better understand the efficacy and safety profile of COLM-SST, real-world evidence was gathered from two separate populations. One was a post-marketing evaluation of open label COLM-SST: The Functional Dyspepsia Adherence and Compliance Trial (FDACT), and the other was an adverse event reporting analysis: The Functional Dyspepsia Safety Update at 36 months (FDSU36). FDACT was conducted to determine real-world daily COLM-SST capsule usage frequency and patient satisfaction in a general population of patients with FD. FDSU36 relied on data gathered from adverse event reports originating from patients using COLM-SST over a 3-year period. In the current analysis, we report the overall satisfaction with symptom relief and usage patterns of COLM-SST users as well as the 36-month safety event profile.

## Methods

### Patient-reported outcomes (PRO) survey study design

FDACT included patients with FD symptoms from the general population who were using open label COLM-SST (marketed as FDgard®, Nestlé HealthCare Nutrition). FDACT participants either received a sample of COLM-SST from their healthcare provider, after evaluation of their symptoms, or purchased COLM-SST in the pharmacy. Those who selected COLM-SST at the pharmacy were not confirmed to have a FD diagnosis prior to participation. Although COLM-SST was available to HCPs and in pharmacies nationwide, geographical distribution of participants was not assessed. An invitation to access the FDACT web-based survey questionnaire was included in COLM-SST packages. The non-validated 10-question survey (Table 1) assessed symptom improvement, patient satisfaction, and dosing frequency in a real-world setting. The questions included frequency of FD symptoms, daily consumption of capsules, timing of consumption of capsules in relationship to meals, time to relief after consuming COLM-SST, improvement in FD symptoms (including EPS and PDS), quality of life, and patient satisfaction. Patients in FDACT were allowed flexible dosing of COLM-SST and had no concomitant medication restrictions. Efficacy was measured using a 3-point Likert scale based on whether there was a major, moderate or no improvement in symptoms following PO-SST use. Assessment was based on a general question related to the overall improvement of FD symptoms after taking COLM-SST and 2 subsequent questions around improvement of abdominal pain and fullness. Although these symptoms represent EPS (abdominal pain) and PDS (fullness), the two were not distinguished separately within a subgroup analysis. First time users versus repeated users were not differentiated in the results of this survey nor were those who selected COLM-SST from the pharmacy versus those who received a sample from their healthcare provider. Patients submitted survey responses anonymously online and received a $2.00 discount coupon for completing the survey. Since this was a patient survey with anonymous responses, IRB approval was not required. In addition, this was not a prospective study so there was no need to register on clinical trials.gov. The survey was conducted between May 2, 2016, and May 15, 2017.

**Table 1:** FDACT Questionnaire

How often do you suffer from persistent or recurrent indigestion (Functional Dyspepsia)?	Daily
	Once a week
	Twice or more a week
	Once a month
On average, how many capsules of FDgard® are you taking?	1–2
	3–4
	5–6
	I don’t take it daily
When do you typically take FDgard®?	Before meals
	After meals
	Only when I have symptoms
How long does it take for you to feel relief from your indigestion after you have taken FDgard®?	Less than an hour
	1–2 h
	3–8 h
	8–24 h
	Longer
Overall, how would you rate the improvement in your functional dyspepsia (indigestion) while taking FDgard®?	Major improvement
	Moderate improvement
	No improvement
Overall, how would you rate the improvement in your quality of life while taking FDgard®?	Major improvement
	Moderate improvement
	No improvement
Overall, how would you rate the improvement in your abdominal pain while taking FDgard®?	Major improvement
	Moderate improvement
	No improvement
Overall, how would you rate the improvement in fullness while taking FDgard®?	Major improvement
	Moderate improvement
	No improvement
How likely are you to recommend FDgard® to family or friends who have Functional Dyspepsia (FD)?	Very likely
	Likely
	Not likely
How likely is it that you will continue taking FDgard® for your FD?	Very likely
	Likely
	Not likely

### 36-month safety data analysis

The safety of COLM-SST was evaluated from July 8, 2016, to July 8, 2019 using data obtained from an independent call center. A call-in number for reporting adverse events (AEs) was provided on all boxes containing COLM-SST. The call center was staffed with pharmacovigilance-trained health care personnel, in accordance with the FDA and global regulatory guidelines on properly reporting AEs, to receive and record customer AEs. The internationally harmonized Medical Dictionary for Regulatory Activities (MedDRA) was used for coding AEs. Version 19 was used for the period between 2016 and 2017. Version 20 was used for the period between 2017 and 2018. Version 21 was used for the period between 2018 and 2019.

## Results

### FDACT

Six hundred patients with FD who either purchased a box of COLM-SST or received a sample of COLM-SST from their health care provider completed the FDACT survey. Demographic information (age, gender) was not collected. Most patients in FDACT reported suffering from FD symptoms daily (75.5%) or twice a week or more (16.7%). Additional survey questions focused on frequency of COLM-SST dosage, symptom relief, and patient satisfaction.

Most respondents (93.8%) in the survey reported that they took COLM-SST on a daily basis. Generally, patients took 1 to 2 capsules per day (47.5%) or 3 to 4 capsules per day (43.7%). Most respondents took COLM-SST in association with consumption of food. More than half (56.2%) indicated that they took the capsules before a meal, while 27.5% indicated that they took it after a meal. Sixteen percent of respondents reported using COLM-SST for on-demand symptom relief.

**Fig. 1:**
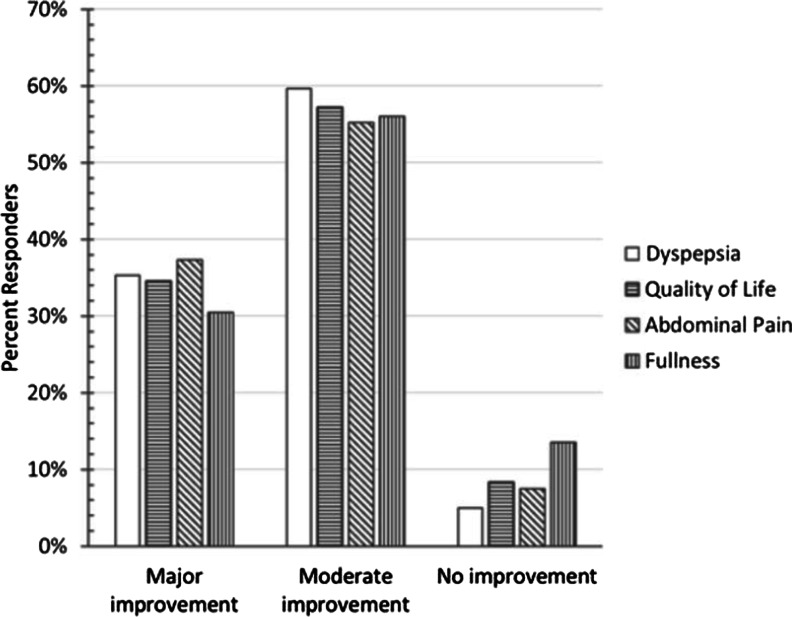
Improvement in symptoms and perceived quality of life with COLM-SST

Most patients indicated that relief of FD symptoms came within 2 h of taking COLM-SST. Achievement of relief within 1 h was reported by 39.2% of respondents, and 47.2% achieved relief between 1 and 2 h. Almost all respondents experienced improvement in FD symptoms while taking COLM-SST, with 35.3% experiencing a major improvement, and 59.7% experiencing a moderate improvement. To further explore symptom relief, patients were asked about the improvement in their upper abdominal pain and epigastric fullness. Ninety-two percent of patients indicated a major or moderate improvement in their upper abdominal pain, while 86.5% indicated a major or moderate improvement in their symptom of epigastric fullness attributable to COLM-SST. Figure 1 displays additional detail on symptom relief and quality of life.

When asked to rate their improvement in QOL while on COLM-SST, 91.7% of respondents indicated a major or moderate improvement with COLM-SST and 97.7% indicated that they would be either likely or very likely to continue taking COLM-SST for their FD symptoms. Furthermore, 95.3% indicated that they would be either likely or very likely to recommend COLM-SST to family or friends.

### FDSU 36—adverse event reporting

The total number of people who were considered to have been unique users of COLM-SST was calculated based on adding the number of sample boxes distributed by physicians and the number of boxes sold through retail distribution. Both numbers were adjusted to reflect an estimate for the samples that became active purchasers and repeat buyers of COLM-SST. Using this framework, it was estimated that over 1 million unique patients tried COLM-SST over the 3-year period of monitoring, thus establishing a denominator estimate for rate calculations.

**Fig. 2:**
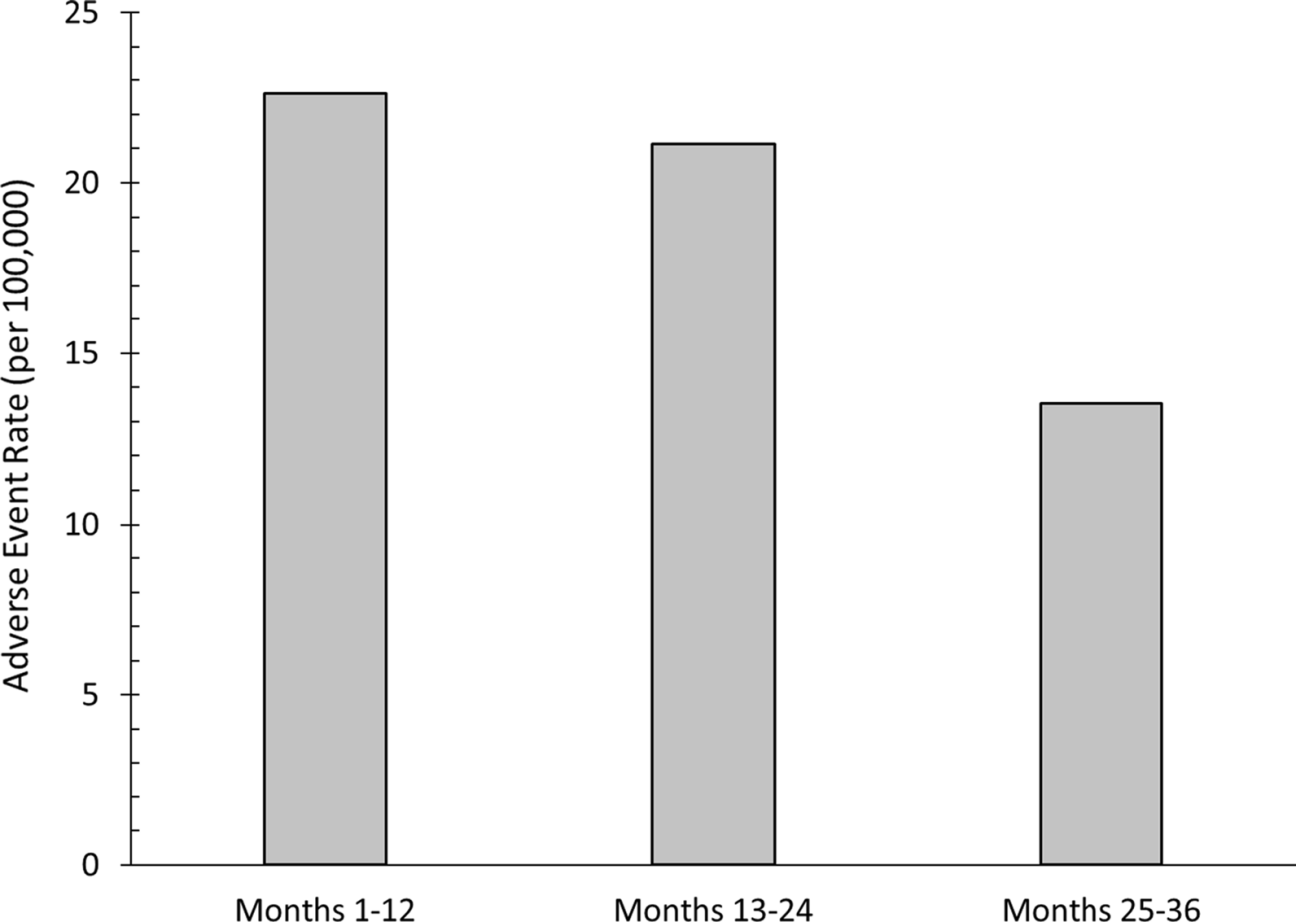
AE rate per 100,000 users of COLM-SST by year

Over the 3-year monitoring period, a total of 205 adverse events were reported to the pharmacovigilance team from 171 Individual Case Safety Reports (ICSRs). No serious adverse events were reported. Of the 205 AEs, 74 were considered unrelated to COLM-SST and 131 were conservatively considered related to COLM-SST due to the temporal relationship of the AE and the consumption of the product and where no meaningful assessment of causality could be established with little to no additional relevant medical history, comorbid condition or concomitant information provided. The 3 most common AEs reported were dyspepsia (n = 22; 12 related and 10 unrelated), diarrhea (n = 13; 9 related, and 4 unrelated), and nausea (n = 11; 5 related and 6 unrelated). Table 2 lists the 10 most common AEs by MedDRA preferred term and Fig. 2 displays the rate of AEs reported by year.

**Table 2:** 10 Most commonly reported Adverse Events associated with COLM-SST*

System organ class	MedDRA term	Cumulative AEs			Individual AEs		
		Months 1–12	Months 1–24	Months 1–36	Months 1–12	Months 13–24	Months 25–36
Gastrointestinal disorders	Dyspepsia	9	10	22	9	1	12
Gastrointestinal disorders	Diarrhoea	4	7	13	4	3	6
Nervous system disorders	Headache	5	9	11	5	4	2
Gastrointestinal disorders	Nausea	3	6	11	3	3	5
Gastrointestinal disorders	Abdominal distension	4	7	10	4	3	3
Gastrointestinal disorders	Abdominal pain upper	3	6	9	3	3	3
Gastrointestinal disorders	Throat irritation	4	6	9	4	2	3
Gastrointestinal disorders	Abdominal discomfort	2	7	8	2	5	1
Gastrointestinal disorders	Abdominal pain	4	7	8	4	3	1
Skin and subcutaneous tissue disorders	Pruritus	1	4	6	1	3	2
**Total AEs**		**75**	**148**	**205**	**75**	**73**	**57**

*Total accumulative sample size (FDgard® users) at 24 months was estimated at 558,300; Estimated total users at 36 months was > than 1,000,000. Adverse Events listed by system organ class and further divided into MedDRA Term

## Discussion

Functional dyspepsia is a highly prevalent and chronic disorder that causes significant patient distress, negatively affects quality of life, and incurs significant costs to the health care system. At present, no medication is approved by the FDA for the treatment of FD. This means that FD patients use lifestyle modifications and/or off-label products (either prescription or over the counter) to manage their FD symptoms, with varying degrees of success [16, 29]. Many of these products are expensive or are associated with side effects. Thus, additional effective, safe, and well tolerated treatment options for FD would be of considerable benefit to patients and providers.

The results from the PRO study FDACT indicate a high level of user satisfaction with COLM-SST as a treatment for the symptoms of FD. We believe that there are multiple possible explanations for this finding. COLM-SST appears to be effective at mitigating or even preventing FD symptoms, with the vast majority of respondents indicating major or moderate FD symptom improvement while taking this therapy. Patient responses indicate that COLM-SST was also effective at relieving both subgroups of FD symptoms according to the Rome IV criteria, those with abdominal pain and/or burning (EPS) and those with fullness or early satiation (PDS). The observations from FDACT support previous clinical trial data from the FDREST study on COLM-SST [26]. Another possible explanation for the high level of satisfaction with COLM-SST is the rapidity of FD symptom improvement, with 86.4% of respondents indicating relief within two hours. This observation also supports the findings from the previous FDREST clinical trial [26]. The PRO data collected in FDACT also suggest a high degree of patient compliance with the directions and use of COLM-SST. It is recommended that COLM-SST be taken daily, 30–60 min before a meal, to maximize effectiveness. The timing of this recommended dosing derives from the observation that FD symptoms frequently worsen in response to eating food [7, 8]. More than half (56.2%) of respondents appeared to follow this recommendation closely, taking the product before a meal, with another 27.5% of respondents taking the product, still in conjunction with a meal, but afterwards. COLM-SST was designed to not only address acute symptoms of FD [30–32], but also to help address chronic gastrointestinal disruption which may contribute to FD symptoms. Menthol for instance, has been shown to have gastroprotective properties [33] and may help to improve nutrient absorption by acting to enhance mucosal absorption [34], while caraway oil along with peppermint oil (whose primary component is L-menthol) have been shown to normalize gastric transit time [32]. As symptoms of disordered gastric emptying have been tied to FD in previous studies, this may provide an explanation for benefit in some FD patients, although this was not directly measured in the current study [35]. Together, we believe the effectiveness of the product, coupled with a high level of patient compliance, are the reasons behind the correspondingly high level of patient satisfaction and self-reported improvements in QOL.

Importantly, no serious AEs have been reported in association with COLM-SST since its launch in 2016. Of the 205 AE reports (out of an estimated 1 million unique users), 74 were subsequently considered to be unrelated to COLM-SST. The remaining 131 AE could not be conclusively linked directly to COLM-SST use but were conservatively included due to the inability to conclusively identify any other cause. Nonetheless, using this most conservative estimate of AE, the COLM-SST rate drops to 0.0119%. This value equates to a number needed to harm of 8,403. Moreover, most AEs reported were associated with symptoms that are either closely related to the condition for which COLM-SST was being taken, or the condition itself. Most reported AEs were considered gastrointestinal in nature; dyspepsia, diarrhea, nausea, and various types of abdominal pain/discomfort. Considering that the population of patients who would use COLM-SST are likely to be suffering from one or more of these symptoms, it is likely that a number of these reports may have been confounded by the underlying condition itself. For example, it is well known that patients with FD frequently have co-existing IBS symptoms [36], and thus it would not be surprising that some patients mistakenly attribute their IBS symptoms as a possible AE. While heartburn would be an expected AE given the L-menthol component in COLM-SST, it was not commonly reported. This may be due to the unique design of COLM-SST, targeted to deliver the L-menthol beyond the pylorus, which helps to minimize the incidence of gastroesophageal reflux symptoms and heartburn. Of note, there were 9 reports of throat irritation during the 36-month monitoring period, and it is possible that patients complaining of throat irritation were in fact experiencing heartburn. Interestingly, while the number of users of COLM-SST increased from an estimated 558,300 in year 2 to over 1 million in the 3-year reporting period, the AE rate decreased.

With the US Congress’ approval in 2016 of the 21^st^ Century Cures Act, there has been an increasingly strident call to include real-world evidence in the evaluation of potential therapies for medical disorders. One of the objectives of this act was to measure and evaluate patient experience information to facilitate more rapid drug and device approval [37, 38]. This type of patient experience includes PROs [39], such as those presented in the FDACT and FDSU36 studies. While not as controlled or rigorous as an RCT, and inherently subject to bias, evidence such as that obtained in these studies, especially with their large sample sizes, can bolster the medical community’s understanding of the efficacy and safety of current and emerging therapies.

The studies reported here are subject to several limitations. Although the data set for the FDSU36 was quite large, the PRO sample size from the FDACT study was small by comparison. Demographic data with a breakdown of age, gender, and race as well as concomitant medication history was not available for either of the FDACT datasets, preventing further analysis of patient characteristics to identify who would most benefit from COLM-SST. Therefore, gathering and analyzing additional data surrounding the real-world experience with COLM-SST is important to better understand who might benefit most from the therapy, and even more importantly, whether it is safe.

Patient generalizability is another potential limitation of these studies. The population of participants for the FDACT study was gathered from individuals who obtained COLM-SST samples from their health care provider or purchased COLM-SST from the pharmacy and then went online to fill out a survey. The survey did not specifically differentiate first time users from repeated users or whether the COLM-SST was obtained from the HCP or pharmacy, therefore contributing to possible bias of subject selection method. Those individuals who might have been regular frequent users may have already had a previous positive experience with COLM-SST and this inherently could bias the results. In addition, the small incentive offered towards future purchase of COLM-SST could be construed as a tacit indicator of patient satisfaction. Future studies are being considered to gather additional PRO data including number of days with symptoms, number of days using COLM-SST, dosing patterns and symptom relief in relation to meals, as well as validated numerical scales to measure quality of life changes before and after using COLM-SST.

## Conclusion

In summary, the data reported here, combined with the previous RCT conducted on COLM-SST [26] provide compelling evidence that COLM-SST is effective at addressing multiple symptoms of FD and is also safe and well tolerated. COLM-SST provides a viable treatment option for patients with FD.

## Data Availability

The datasets used and/or analyzed during the current study are held electronically by Survey Monkey Inc. presently known as Momentive Inc**.** Survey Data is available from the corresponding author on reasonable request.

## Abbreviations

FD: Functional Dyspepsia
DGBI: Disorder Gut Brain Interaction
EPS: Epigastric Pain Syndrome
PDS: Post-prandial Distress Syndrome
FDACT: Functional Dyspepsia Adherence and Compliance Trial
FDSU 36: Functional Dyspepsia Safety Update at 36 months
COLM-SST: Caraway Oil L-Menthol Site Specific Targeting
PRO: Patient Reported Outcomes
AE: Adverse Events
QOL: Quality of Life
MedDRA: Medical Dictionary for Regulatory Activities
FDA: Food and Drug Administration
RCT: Randomized Control Trial
